# Gill Transcriptome Sequencing and De Novo Annotation of *Acanthogobius ommaturus* in Response to Salinity Stress

**DOI:** 10.3390/genes11060631

**Published:** 2020-06-08

**Authors:** Zhicheng Sun, Fangrui Lou, Yuan Zhang, Na Song

**Affiliations:** Key Laboratory of Mariculture, Ocean University of China, Ministry of Education, Qingdao 266003, China; sunzhicheng@stu.ouc.edu.cn (Z.S.); 11170511026@stu.ouc.edu.cn (F.L.); 11180511034@stu.ouc.edu.cn (Y.Z.)

**Keywords:** *Acanthogobius ommaturus*, RNA-seq, salinity stress, differentially expressed genes (DEGs)

## Abstract

*Acanthogobius ommaturus* is a euryhaline fish widely distributed in coastal, bay and estuarine areas, showing a strong tolerance to salinity. In order to understand the mechanism of adaptation to salinity stress, RNA-seq was used to compare the transcriptome responses of *Acanthogobius ommaturus* to the changes of salinity. Four salinity gradients, 0 psu, 15 psu (control), 30 psu and 45 psu were set to conduct the experiment. In total, 131,225 unigenes were obtained from the gill tissue of *A. ommaturus* using the Illumina HiSeq 2000 platform (San Diego, USA). Compared with the gene expression profile of the control group, 572 differentially expressed genes (DEGs) were screened, with 150 at 0 psu, 170 at 30 psu, and 252 at 45 psu. Additionally, among these DEGs, Gene Ontology (GO) analysis indicated that binding, metabolic processes and cellular processes were significantly enriched. Kyoto Encyclopedia of Genes and Genomes (KEGG) pathways analysis detected 3, 5 and 8 pathways related to signal transduction, metabolism, digestive and endocrine systems at 0 psu, 30 psu and 45 psu, respectively. Based on GO enrichment analysis and manual literature searches, the results of the present study indicated that *A. ommaturus* mainly responded to energy metabolism, ion transport and signal transduction to resist the damage caused by salinity stress. Eight DEGs were randomly selected for further validation by quantitative real-time PCR (qRT-PCR) and the results were consistent with the RNA-seq data.

## 1. Introduction

The salinity is one of the most significant factors for regulating distribution, abundance and diversity of aquatic animals [1], and it is also one of the main environmental factors that exerts selective pressure on aquatic organisms. The sea surface salinity would rise with the increase of temperature under global warming [2] Changes in environmental salinity will directly affect osmotic pressure of aquatic organisms [3], which has been predicted to have a profound impact on fish at the molecular and cellular level. Fish need to make adaptive changes to maintain physiological function, and conduct compensatory adjustment through changing distribution range [4]. 

*Acanthogobius ommaturus* is a large, demersal fish of the Gobiidae, which is economically important across its distribution area [5]. It is a euryhaline fish that could adapt rapidly and maintain homeostasis in a wide range of salinities (from freshwater to salinity 30) [6]. Studies on *A. ommaturus* mainly focused on the comparative analysis of individual fecundity and morphology [7], fishery biology [8], name of species [9], and genetic diversity and structure by molecular markers [10]. However, the potential mechanism of salinity adaptation in *A. ommaturus*, especially at the molecular level, such as comprehensive molecular pathway response, has not been well studied. It is worth noting that the *Acanthogobius flavimanus*, which is closely related to the *A. ommaturus* and distributed in East Asia, has invaded California, USA [11,12] and southern Australia [13]. As a euryhaline fish with strong viability and rapid growth, *A. ommaturus* may be also capable to invade other waters leading to the imbalance or collapse of the local marine ecosystem. Therefore, it is very necessary to conduct the potential mechanism of salinity adaptation of *A. ommaturus*.

With the development of high-throughput sequencing technology, RNA-seq provides an opportunity to study transcriptome variation in organisms under different environmental conditions. Transcriptome sequencing can not only detect almost all effective genes expressed in specific cells or organs, but also provide a comprehensive understanding of the regulatory mechanisms involved in specific biological processes based on the structure and function of differential genes [14]. In addition, transcriptome sequencing allows simultaneous analysis of all physiological process, including metabolism [15], protein homeostasis [16] and other regulatory cellular processes [17]. Thus, transcriptome technology addresses key gene expression patterns of non-model organisms in specific environments and enables detection of unknown genes and discovery of new transcripts. In recent years, transcriptome technology has been widely used in transcriptome assembly and annotation of many non-model fish, such as *Megalobrama amblycephala* [18], *Pelteobagrus fulvidraco* [19] and *Atlantic salmon* [20].

In this study, to further know about the osmoregulatory mechanisms of *A. ommaturus* in response to salinity stress, RNA-Seq was used to sequence transcripts of gill tissues of *A. ommaturus* under salinity stress. This is the first transcriptome study of *A. ommaturus*, which will provide important information for enriching its genetic resources. Moreover, the main purpose of this study is to explore and identify the genes and pathways of gill organs that play a potential role in salt tolerance by using RNA-Seq.

## 2. Materials and Methods

### 2.1. Ethics Statement

All animal experiments were conducted in accordance with the guidelines and approval of the respective Animal Research and Ethics Committees of Ocean University of China. The field studies did not involve endangered or protected species. In addition, frost anesthesia was performed to minimize the suffering of all *A. ommaturus* specimens.

### 2.2. Experimental Design and Sampling

*Acanthogobius ommaturus* specimens (initial weight 41.88 ± 5.52 g, initial length 19.23 ± 1.91 cm) were collected from the coastal water of Qingdao, China. All specimens were dispatched into four aquariums (80 × 60 × 40 cm) with recirculating aerated natural seawater (25 psu) at a density of 20 fishes per aquarium for one week of acclimation. During the acclimation and experimental periods, *A. ommaturus* was fed to satiation with small shrimp twice daily (08:30 and 16:00), and the remaining feed and feces were siphoned out and 1/3 of the water in each aquarium was replaced with pre-aerated water each day. After 7 days of acclimation, healthy fish with strong individual vitality were used for salinity stress experiments. In the manner of acute stress, each box was a salinity gradient, and the four salinity gradients were 0 psu, 15 psu, 30 psu and 45 psu, respectively. In this experiment, since the optimum salinity range of *A. ommaturus* is 5–15 psu [21], salinity 15 was selected as the control salinity. Three groups of experiments with salinity of 0 vs. 15, 15 vs. 30 and 15 vs. 45 were analyzed. After 48 h salinity stress, three experimental individuals with similar size, vigor and health were immediately euthanized and sampled in each experimental group. Gills tissues were collected and stored at −80 °C for RNA extraction.

### 2.3. RNA Isolation and Illumina Sequencing

Total RNA was extracted from the gill tissues using the TRIzol reagent (Invitrogen, Carlsbad, USA) according to the instructions, and genomic DNA was removed using DNase I (TaKaRa, Otsu, Japan). RNA purity was checked using the NanoPhotometer^®^ spectrophotometer (IMPLEN, Calabasas, CA, USA). RNA concentration was measured using Qubit^®^ RNA Assay Kit in Qubit^®^ 2.0 Flurometer (Life Technologies, Carlsbad, CA, USA). RNA quality was determined by an Agilent 2100 Bioanalyzer. Only high-quality RNA samples (OD260/280 = 1.9–2.1, OD260/230 ≥ 2.1, RIN ≥ 9.5, 28/18S ≥ 1.0, > 30 μg) were used to construct these quenching libraries. The RNAs from tissues of salinity gradient were pooled in equal amounts and mRNA was extracted from the total RNA using magnetic beads with Oligo (dT) probes. Fragmentation buffer was applied to lyse the mRNA into fragments with a suitable size and the fragmented mRNA was used to construct a cDNA library using TruSeq Stranded mRNA LTSample Prep Kit (Illumina, San Diego, CA, USA). Then, the sequencing was carried out by Novogene Company (Beijing, China) on Illumina Hiseq 2000 platform. 

### 2.4. De Novo Assembly and Functional Annotation

All raw reads in FASTQ format quality control relied on NGS QC ToolKit software (v2.3.3, National Institute of Plant Genome Research, New Delhi, India) [22] and clean reads were obtained by removing raw reads containing adapter, ploy-N (N ratio > 10%) and low quality reads (quality scores ≤ 5). The Q20, Q30, GC (Guanine and Cytosine)-content and sequence duplication level of the clean data were calculated. All the downstream analyses were based on clean data with high quality. Transcriptome de novo assembly was carried out with Trinity software (version 2.0.6 Broad Institute, Cambridge, MA USA) with a min_kmer_cov set to 2 by default and all other parameters set to their defaults [23]. After assembly, the transcripts were clustered and the redundant ones were removed and the remaining sequences were defined as unigenes. All of the assembled unigenes were searched against the Nr (NCBI non-redundant protein sequences) database, the eukaryotic orthologous group (KOG) database, COG (Clusters of Orthologous Groups of Proteins) database, Pfam (Protein family) database, SwissProt databases, the Kyoto Encyclopedia of Genes and Genomes (KEGG) database and Gene Ontology (GO) database using BLASTx to identify the proteins that had the highest sequence similarity with the given transcripts to retrieve their function annotations, and a typical E-value cut-off was set at < 1.0 × 10^−5^ [24].

### 2.5. Differential Expression Analysis

To investigate the different gill transcriptome responses of *A. ommaturus* to salinity fluctuation, we analyzed the number and biological functions of differentially expressed genes (DEGs) in three treatment groups. Firstly, we used BWA-mem [25] to map all unigenes to a multicast file. Then, the expression levels of all unigenes were normalized using RSEM [26] and Bowtie2 [27] to determine the number of fragments per kilobase of exon model per million mapped fragments (FPKM). DEGs of three treatment salinity were identified using the edgeR package [28], and |log_2_FC| ≥ 1 (which corresponds to FC = 2) and FDR ≤ 0.05 were used as the filtering thresholds. In addition, in order to further understand the function of DEGs under salinity stress, DEGs were used to determine the Gene Ontology (GO) term and the Kyoto Encyclopedia of Genes and Genomes (KEGG) pathway. Blast2GO (https://www.blast2go.com/) software was used to obtain the GO annotations of unigenes for describing biological processes, molecular functions and cellular components [29]. The KEGG Automatic Annotation Server (KAAS) (http://www.genome.jp/kaas-bin/kaas_main) system was used for pathway reconstruction, respectively [30].

### 2.6. Quantitative Real-Time PCR

In order to verify the reliability of the transcriptome sequencing results, eight differentially expressed genes (DEGs) with high expression levels were randomly selected, and primers were designed with Primer 6.0 (Table S1). Firstly, reverse transcription was carried out from each total RNA sample using the PrimeScript RT reagent Kit with gDNA Eraser (TaKaRa, JPN) with RT Primer Mix of Random 6 mers and Oligo dT Primer. Furthermore, standard curves were constructed to identify the ideal dilution times of cDNA samples and were used as calibrators. A total of 12 cDNA samples were diluted in 10-fold with nuclease-free water and were used as templates for PCR. Furthermore, the qRT-PCR analysis was designed following instructions for SYBR^®^ Premix Ex TaqTM (Tli RNaseH Plus) RR420A with a StepOnePlus (TaKaRa, JPN). A reaction system of 20 μL was amplified, including 2.0 μL of diluted cDNA template, 10μL of SYBR Premix Ex Taq (2×), 0.4 μL of each of the forward and reverse primers and ROX Reference Dye (50×) and 6.8 μL of nuclease-free water. The amplification processes consisted of a holding stage of 30 s at 95 °C, followed by 40 cycles of 5 s at 95°C and 35 s at 52 °C. All reactions were performed in triplicates. Melting curve analysis was performed to determine the target specificity, and melting curve temperature was 61.9–86.7 °C. The relative expression levels of all target unigenes were calculated by the 2^−ΔΔCT^ analysis method (ΔCT = CT_target unigene_ − CT_reference gene_, ΔΔCT = ΔCT_treatment_ − ΔCT_control_), and β-actin was used as the reference gene for qRT-PCR normalization.

### 2.7. Statistical Analysis

Statistical analyses were performed using SPSS 18.0 software. Data expressed as the mean ± standard deviation (SD) from three independent replicates. Significant differences between samples were analyzed by Duncan’s tests at a significance level of 0.05.

## 3. Results

### 3.1. Illumina Sequencing and Assembly

In brief, RNA-Seq was carried out on libraries constructed from gill of *A. ommaturus*. After quality trimming and adapter clipping, results from RNA-Seq produced 12 libraries that consisted of 638,902,551 clean reads with an N50 value equal to 1111. The error rate was less than 0.03, Q30% (the rate of bases for which quality is greater than 20) was over 93%, and GC percentage was around 47–49%, indicating high-quality sequencing and the feasibility of the subsequent analyses. After de novo assembly using trinity software (version 2.0.6 Broad Institute, Cambridge, MA, USA), 131,225 unigenes and 88,228,207 assembled bases were assembled. The overall sequencing results are shown in Table S2. All raw sequence data were deposited in NCBI Sequence Read Archive (SRA) under accession number SRP258637.

### 3.2. Unigenes Functional Annotation and Classification

Unigenes were subjected to annotation analysis by comparing with Nr, KOG, COG, SwissProt, Pfam, KEGG, and GO databases. Results show that a total of 46,443 unigenes (35.38%) were annotated in at least one database, with 39,920 annotated unigenes (30.42%) had a significant BLAST hit against Nr database. The detailed annotation results are listed in Table 1. For top-hit species matched against Nr database, 51.4% of the matched unigenes indicated similarity with *Boleophthalmus pectinirostris* (51.4%), followed by *Larimichthys crocea* (3.8%) and others (31.9%) (Figure S1).

### 3.3. Analysis of Differentially Expressed Genes (DEGs)

**Figure 1:**
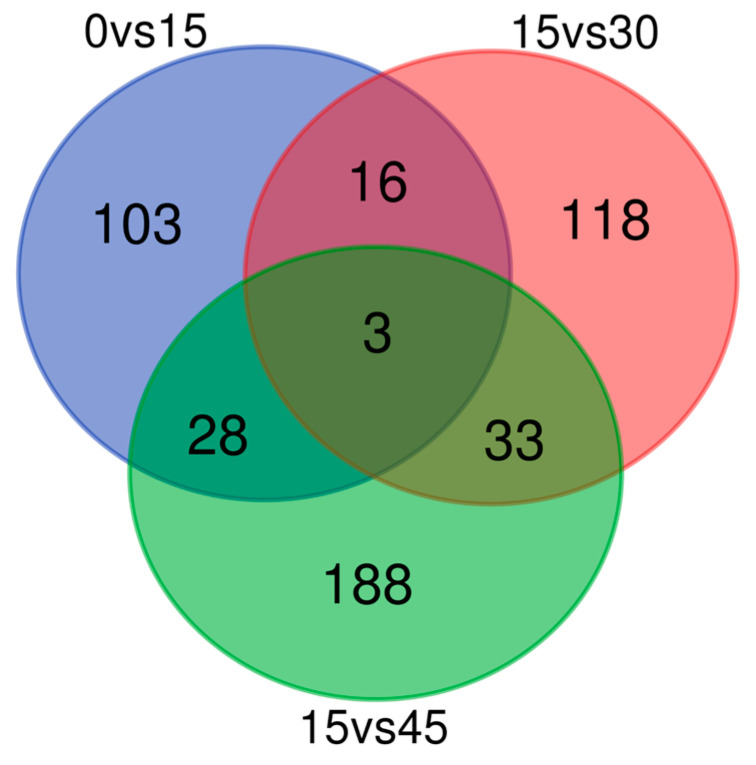
Global overview of differentially expressed unigenes based on Venn diagram.

To elucidate the gene expression pattern under salinity fluctuation, we firstly compared the numbers of DEGs of three treatment groups. A total of 150 (59 up- and 91 down-regulated), 170 (39 up- and 131 down-regulated), and 252 (159 up- and 93 down-regulated) genes were differently expressed with |log_2_FC|≥1 (which corresponds to FC = 2) and FDR ≤ 0.05 in 0 vs. 15 psu, 15 vs. 30 psu and 15 vs. 45 psu, respectively. As shown in Figure 1, three DEGs were present in all three comparisons.

### 3.4. GO and KEGG Pathway Enrichment Analysis of DEGs

**Figure 2:**
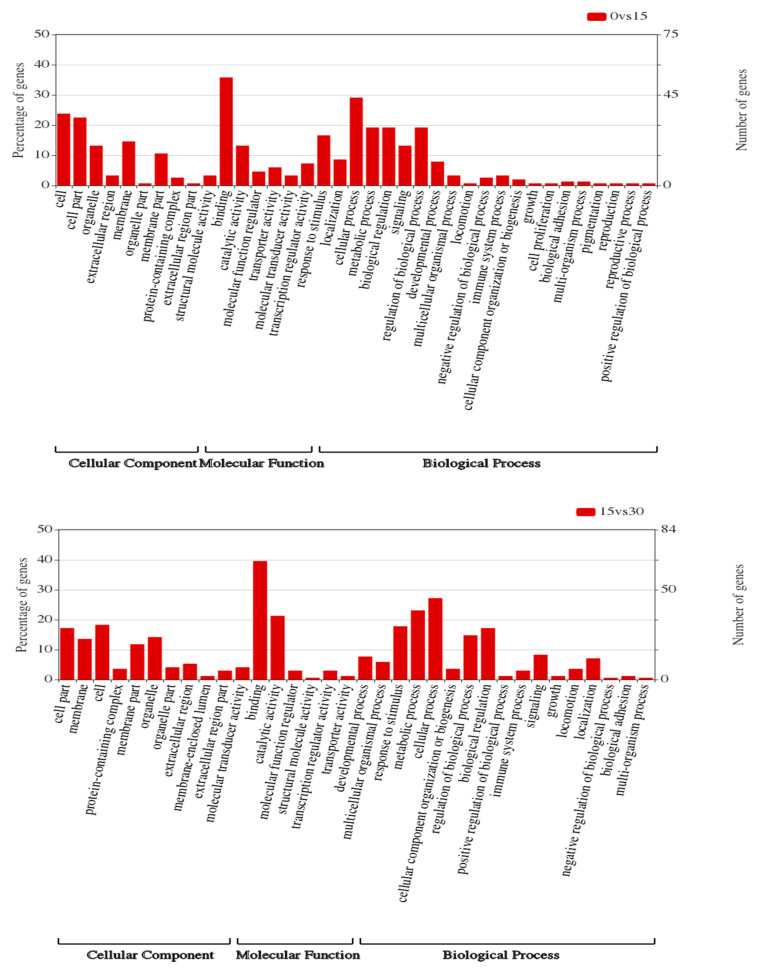

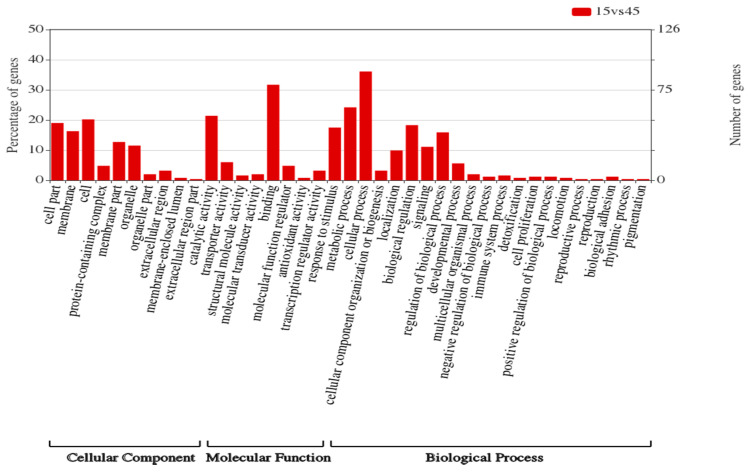
Results of the enrichment analysis of gene ontology (GO) terms of differentially expressed genes (DEGs) of *A. ommaturus* exposed to salinity changes.

To identify the functional changes potentially associated with the salinity changes of *A. ommaturus*, GO and KEGG pathway enrichment analyses were conducted. Gene ontology analysis of the three comparisons was performed to ascribe GO categories to 150, 170 and 252 DEGs, respectively. Compared to the control group (15 psu), 101 of 150, 109 of 170 and 167 of 252 significant DEGs were classified into 37 GO terms, 34 GO terms and 39 GO terms at 0 psu, 30 psu and 45 psu, respectively. The distribution of GO terms indicated that cellular processes (GO: 0009987) and metabolic processes (GO: 0008152) in biological processes, cells (GO: 0005623) and cell parts (GO: 0033643) in cellular components, and binding (GO: 0005488) and catalytic activity (GO: 0003824) in molecular functions were most significantly enriched under salinity stress. The detailed information regarding significant GO terms is shown in Figure 2. 

**Figure 3:**
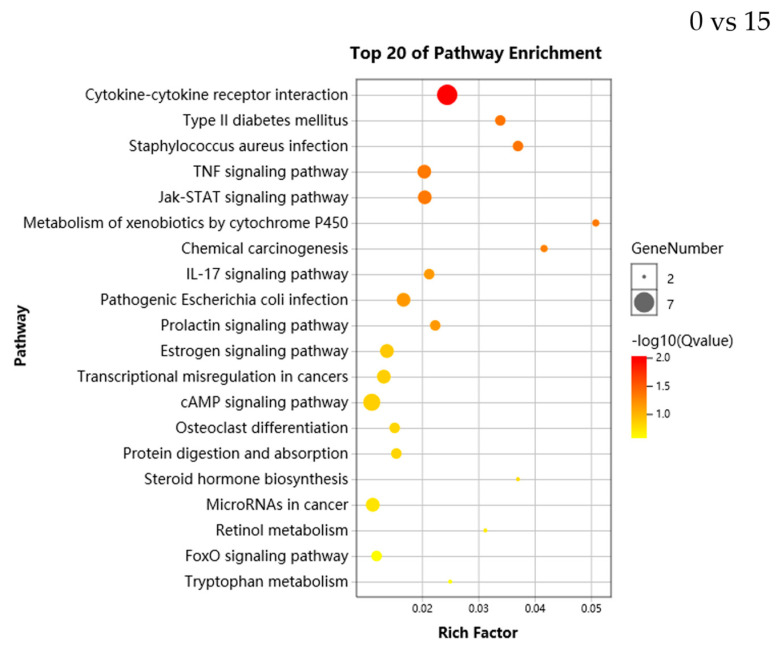

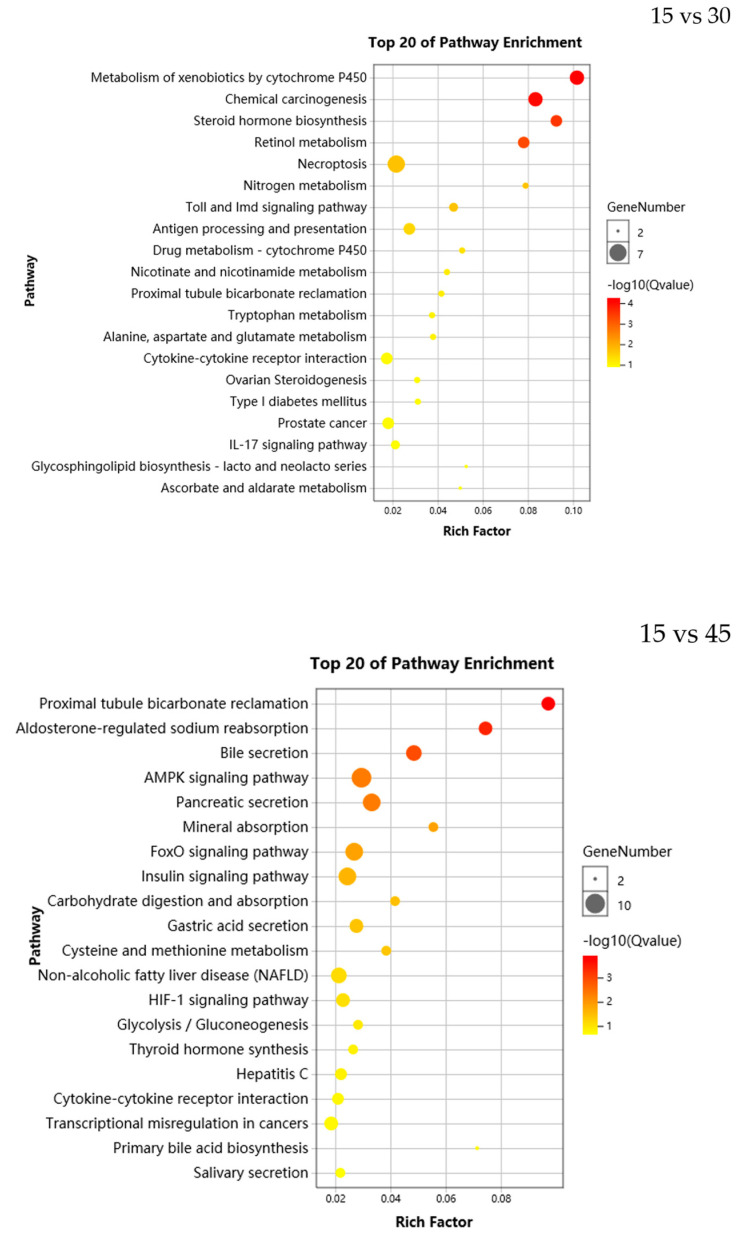
Scatterplot of Kyoto Encyclopedia of Genes and Genomes (KEGG) pathways enriched in the DEGs. Rich factor is the ratio of the DEGs number to the total gene number in a given pathway. The size and color of the dots represent the gene number and range of the *q* value, respectively.

KEGG pathways provide valuable information for studying the specific biological, metabolic processes and molecular mechanisms under salinity stress in *A. ommaturus*. With the increase of salinity, DEGs were enriched in 170, 168 and 234 different pathways, among which 7, 9 and 11 pathways (*q* value < 0.05) were significantly enriched in 0 vs. 15, 15 vs. 30 and 15 vs. 45 groups, respectively. Compared to the control group, most DEGs were enriched in signal transduction pathways, metabolic pathways, digestive and endocrine systems at 0 psu, 30 psu and 45 psu, respectively. For the KEGG pathway analysis, the dominant pathways were as follows: cytokine–cytokine receptor interaction (ko04060), metabolism of xenobiotics by cytochrome P450 (ko00980) and proximal tubule bicarbonate reclamation” (ko04964) in 0 vs. 15, 15 vs. 30 and 15 vs. 45, respectively. The significantly enriched pathways in the top 20 were shown in Figure 3.

### 3.5. Identification of DEGs Related to Salinity Changes

As a non-model species with only limited gene function annotation resources, we based Nr annotation and manual literature searches on the combination of enrichment analysis to further discuss candidate DEGs potentially associated with salinity adaptation and osmoregulation. These DEGs were categorized into three functional categories including energy metabolism, ion transporters and signal transduction (Table 2) by GO enrichment analysis. Imputed putative functions of these genes are covered in the discussion. 

### 3.6. Validation of Transcriptomic Data by qRT-PCR

**Figure 4:**
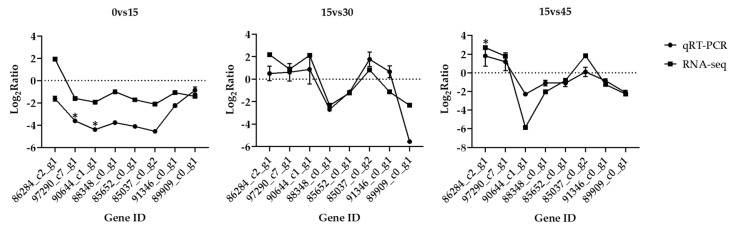
Relative change of the transcriptomes data and qRT-PCR data of 8 DEGs. Log_2_fold changes are expressed as the ratio of gene expression after normalization to β-actin. The data are means ± SD from three independent replicates, and “*” indicates statistical significance between experimental group and control group (*p* < 0.05).

Eight differentially expressed genes (DEGs) were randomly selected to validate the reliability of the transcriptome in this study. Melting-curve analysis revealed a single product for all of the tested genes. For these candidate genes, the variation trend in expression was concordant between qRT-PCR data and transcriptomic data, excluding two genes (86284_c2_g1 in 0 vs. 15 group and 91346_c0_g1 in 15 vs. 30 group) did not match perfectly. Therefore, the results indicated the transcriptomic data were credible (Figure 4).

## 4. Discussion

Salinity is one of the decisive environmental factors affecting fish survival and growth [31], food intake [32], disease [33] and distribution [34]. However, limited evidence is available to explain how salinity stress affects the cellular physiological effects of *A. ommaturus*. RNA-Seq analysis is considered to be a robust method for evaluating transcriptional responses to different experimental conditions, especially in non-model organisms without reported genomes [35]. In this study, 156, 165, 167 and 151 million clean reads were obtained for four salinity gradients of 0 psu, 15 psu, 30 psu and 45 psu, respectively. For most eukaryotic transcriptomes, sequencing up to 100 million reads can quantify precisely genes and transcripts that have low expression levels [36]. According to the analysis of RNA sequencing data, Q30 is more than 93%, and the N50 length of unigenes is 1111 bp, which proves the reliability of raw data. From the distribution of species annotated by nr database, 20,505 of 39,920 unigenes (51.4%) have been annotated to the species of *Boleophthalmus pectinirostris*. This is probably due to the closer phylogenetic relationship between two species since both are in the order of Gobioidei, which is also supported the accuracy of raw data. In addition, to further confirm gene expression profiles, qRT-PCR analysis of eight selected DEGs was conducted. Although there were some differences in gene expression between qRT-PCR and RNA-Seq, the overall gene expression trends were consistent. In conclusion, the sequencing results of this study are accurate and reliable, which is suitable for further analysis.

To further explore the research results, we performed GO and KEGG enrichment analysis of differential expressed genes (DEGs). The results of GO enrichment analysis indicated that DEGs were significantly enriched in binding (GO: 0005488), metabolic processes (GO: 0008152), and catalytic activities (GO: 0003824). Previous studies have confirmed these three functions play an important role in osmoregulatory mechanisms. Firstly, the 14-3-3 protein inhibit Ca^2+^ activated Cl^−^ channels by binding (GO: 0005488) to calmodulin to maintain osmotic pressure homeostasis in the euryhaline teleost *Fundulus heteroclitus* [37]. *Acipenser brevirostrum* appeared to have successfully adapted to higher salinity, probably due to the osmotic pressure balance maintained by the metabolism (GO: 0008152) of non-esterified fatty acids [38]. In addition, previous studies had shown that the catalytic function (GO: 0003824) of enzymes regulated ion changes and osmotic pressure when crustaceans were exposed to salinity fluctuations [39]. KEGG pathway analysis can enhance our understanding of how salinity fluctuations affect the osmoregulation of *A. ommaturus*. For the 0 vs. 15 group, cytokine receptor interaction and JAK STAT signaling pathway related to signal transduction was upstream pathways regulating immune and osmoregulation [40]. Steroid hormone biosynthesis and amino acid metabolism associated with metabolic pathways involved in osmoregulation were significantly enriched in the 15 vs. 30 group. Steroid hormone biosynthesis in metabolic pathways played pivotal roles in response to salinity stress and was inextricably linked to other pathways in aquatic animals [41]. The variation of amino acids will directly lead to the change of protein content [38], which facilitates more rapid acclimation to environmental changes than relying solely on enzymatic mechanisms [42]. Regarding the annotated path map (Figure S2) in the 15 vs. 45 group, pancreatic secretion, mineral absorption, proximal tubule bicarbonate reclamation and bile secretion have a common feature of increasing the ATPase or ATP content to provide energy for the exchange of sodium and potassium ions. In these pathway maps, we found high expression of genes involved in osmotic pressure regulation, such as cystic fibrosis transmembrane conductance regulator (*CFTR*), Na^+^-K^+^-2Cl^-^ cotransporter 1 (*NKCC1*), and phosphoenolpyruvate carboxykinase (*PEPCK*). In addition, the energy metabolism, ion transporters and signal transduction related DEGs and their potential functions of salinity response were discussed below.

### 4.1. DEGs Related to Energy Metabolism

In order to maintain osmotic balance, the osmoregulatory processes are regulated by several kinds of enzymes, and the synthesis and operation of these proteins requires large amounts of energy [4]. Stress induced by salinity changes has been associated with enhanced reactive oxygen species (ROS) generation, causing oxidative damage [43]. Glutathione S-transferase (*GST*) is a key enzyme of cellular detoxification systems that defends cells against ROS [44]. However, compared with the control salinity, *GST* was down-regulated at 0 psu and 30 psu in this study. These differences in *GST* response may highlight differences in detoxification capacity between species and different tissues [45]. We hypothesized that the down-regulated expression of *GST* at 0 psu and 30 psu was related to the salinity tolerance of *A. ommaturus*. Glutamine synthetase (*GS*) activity may facilitate the production of glutamine, proline, and other organic solutes characteristic of osmotic and pH adjustments under stress conditions [46]. *GS* expression was significantly up-regulated under high salinity stress (30 psu and 45 psu). We speculated that the high expression of *GS* promoted the production of proline and other amino acids, which was widely used in hyperosmotic fermentation systems as an osmotic pressure protectant [47]. It is worth noting that phosphoenolpyruvate carboxykinase (*PEPCK*) was up-regulated at 30 psu. *PEPCK* catalyzes the conversion of oxaloacetate into phosphoenolpyruvate, which is the first rate-limiting step in gluconeogenesis [48]. The increased expression of *PEPCK* in the gills of *A. ommaturus* indicated that the endogenous glucose increased by enhancing the gluconeogenesis to provide the extra energy to maintain the ion balance under the change of salinity [48]. In brief, we hypothesized that genes associated with energy metabolism provided energy for cell survival and ultimately increased the ability of *A. ommaturus* to survive in salinity changes.

### 4.2. DEGs Related to Ion Transporters

Osmotic balance is maintained by the coordinated transport between water and ions in the intestines, gills and kidneys in teleosts [49]. The gill is an osmoregulartory organ; several classical ion transporters and channels were found in this study, such as Aquaporin (*AQP1* and *AQP3*), transient receptor potential cation channel subfamily V member 4 (*TRPV4*), Na^+^-K^+^-2Cl^-^ cotransporter 1(*NKCC1*), ATP-sensitive inward rectifier potassium channel 15(*KCNJ15*) and sodium potassium ATPase beta subunit (*NKA*) [50,51,52,53]. Tilapia is one of the typical experimental models for euryhaline fish. It would be more informative if we discuss the similarity and differences in the patterns of gene expression of gill ion transporters and channels between tilapia and *A. ommaturus*. To acclimate in a high salinity environment, *NKA* transports 3Na^+^ outward in exchange for 2K^+^, creating low intracellular Na^+^ and a highly negative charge within the cell; the Na^+^ gradient is used to transport Na^+^, K^+^ and 2Cl^−^ into the cell through a basolateral *NKCC1*; Cl^−^ then leaves the cells down an electrical gradient through an apical *CFTR* [54]. In this osmoregulatory mechanism, the up-regulation of *NKA*, *NKCC1a* and *CFTR* in the gills of *A. ommaturus* does make sense; the similarity in the patterns of gene expression in *Oreochromis mossambicus* [55] adapted to seawater stress. The currently accepted model for active NaCl secretion by ionocytes in seawater-acclimated teleosts consists primarily of the cooperative action of three major ion-transport proteins: *NKA*, *NKCC1*, and *CFTR* [54]. Under low salinity environment, the basolateral *NKA* activity is probably involved in driving uptake of NaCl, possibly in conjunction with an apical V-type H^+^-ATPase, via apical Na^+^ channels (90406_c5_g1, log_2_FC = 0.231) and Cl^–^/HCO_3_^–^ exchangers [56]. The up-regulation of *NKA* plays an important role in freshwater adaptation of *A. ommaturus* and *O. mossambicus* [55]. In the same way, the up-regulation of *NKA* has been found in *Salvelinus namaycush* and *Salvelinus fontinalis* [57], and in *Stenogobius hawaiiensis* [50] in both high and low salinity environments. *NKA* provides the major driving force for both ion secretion and absorption [58], this may be closely related to the up-regulated expression of *NKA* in both high and low salinity environments. 

Teleost fishes are able to acclimatize to seawater by secreting excess NaCl by means of specialized “ionocytes” in the gill epithelium [59]. The main function of *NKCC* is ion secretion [59,60], which explains that the up-regulation of *NKCC* under high salinity environment and down-regulated under low salinity environment in *A. ommaturus*. Unlike *A. ommaturus*, the up-regulation of *NKCC* in *O. mossambicus* occurred during freshwater adaptation, which was surprising [55]. Moreover, ion absorption function Na^+^/Cl^–^ cotransporter (*NCC*) and Na^+^/H^+^ exchanger 3 (*NHE3*), were up-regulated in freshwater adaptation of *O. mossambicus* [58,60], but this significant phenomenon was not found in *A. ommaturus*. Although the expression of *NHE3* in *A. ommaturus* was up-regulated in the low salinity environment, the log_2_FC value was only 0.167. The similarity and differences in the patterns of gene expression in salinity adaptation between *A. ommaturus* and *O. mossambicus* suggests the likelihood that there is more than one type of ionocyte in most teleost fishes adaptable to freshwater, while ion-secretory ionocytes in seawater seem to be of only one type in all teleosts [55]. Prolactin (*PRL*) is a freshwater adapting hormone in teleosts that acts by reducing water permeability and promoting ion uptake by osmoregulatory organs, such as gills [61]. *PRL* release in a hypoosmotic environment is induced by an increase in *PRL* cell volume brought about by the influx of water via a membrane-bound *AQP3* [62] and Ca^2+^ influx through the stretch-activated channel *TRPV4* [63]. *PRL* may play an important role in the control of water and electrolyte balance through *PRLR* expressed in the osmoregulatory organs in the marine teleost [64]. Despite, the significantly up-regulated expression of *PRLR* instead of *PRL*, we suspected that the up-regulated expression of *AQP3* and *TRPV4* at 0 psu were the direct reason that promoted the secretion of *PRL* and led to the up-regulated expression of *PRLR* to adapt to freshwater stress. We found that the expression patterns of *AQP3* and *TRPV4* genes in *A. ommaturus* and *O. mossambicus* were similar, both of which were up-regulated under freshwater stress [62,63]. In addition, *AQP1* in gills of *Acanthopagrus schlegeli* [65] and *Dicentrarchus labrax* [66] decreased with the increase in salinity, which was consistent with the results of this experiment. In the same way, *FXYD* also plays an important role in osmoregulation [67]. In general, the patterns of gene expression of osmoregulatory mechanisms between *A. ommaturus* and *O. mossambicus* were largely similar. The synergistic effect of ion transportations and channels elicits complex regulatory strategies and ultimately increases tolerance to salinity stresses.

### 4.3. DEGs Related to Signal Transduction

The adaptability and stimulatory response of fish to salt stress depends on the effective mechanism of osmosensing and osmotic stress signaling [68]. Furthermore, osmosensory signals are not only rapidly transduced within cells, but may also be amplified and distributed to many types of downstream osmotic effects [68,69]. The cAMP as a signal factor participates in the regulation of osmotic pressure by mediating the increase of Na^+^ uptake by cells in the internal environment [70]. At the same time, the cAMP signaling pathway stimulates the production of arachidonic acid metabolites, which regulates the production of cortisol and glucagon, osmotic regulation and cellular fatty acid signaling in fish [71]. The “cAMP-responsive element modulator (*CREM*)” and “cAMP-specific 3′, 5′-cyclic phosphodiesterase 4B (*PDE4B*)” were both down-regulated at 0 psu and up-regulated at 45 psu. We suspected that the down-regulated expression of *CREM* and *PDE4B* at 0 psu inhibits Na^+^ uptake and increases water transport, which maintains the balance of osmotic pressure. On the contrary, the up-regulation expression of *CEMR* and *PDE4B* at 45 psu enhances metabolic and osmolarity-regulated signals, providing energy and signaling mechanisms for maintaining osmolarity balance. Strangely enough, high affinity cAMP-specific and IBMX-insensitive 3′, 5′-cyclic phosphodiesterase 8A did decrease at 30 psu, whether the specific mechanism of action related to the salinity tolerance of 30 psu is yet to be further explored. Furthermore, prolactin receptor (*PRLR*) was up-regulated at 0 psu for *A. ommaturus*. For freshwater adaptation, prolactin (*PRL*) is known to reduce Na^+^ efflux and water permeability, and is up-regulated under freshwater stress (0 psu) [72], and *PRL* initiates its role by binding to a specific cell surface *PRL* receptor (*PRLR*) [73]. In this way, *PRLR* participates in osmotic pressure regulation by affecting the binding of *PRL* hormones, and it indirectly regulates sodium ions to maintain the stability of osmotic pressure [64]. It is predicted that all teleost species may have the *PRL*/*PRLR* system to adapt to sudden osmotic changes in the environment, and that *PRL*s are widely expressed in osmotically regulated organs [64]. These signals have important functions in the signal cascade of osmotic regulation. It can be used to amplify or transmit osmotic sensory signals and then regulate the functional characteristics of various downstream osmotic effects.

## 5. Conclusions

In this study, RNA-seq analysis was performed on *A. ommaturus* exposed to four salinity gradients (0 psu, 15 psu, 30 psu, and 45 psu), and the obtained high quality data could be the best guarantee of the reliability of experimental results. After the verification of this experiment, these changes are related to metabolic processes, ion transport, signal transduction and other characteristics. We also found that the similarity in the patterns of gene expression of gill ion transporters and channels in salinity adaptation between *A. ommaturus* and *O. mossambicus*. Compared with high salinity stress, *A. ommaturus* is better able to adapt to low salinity stress. This information will improve our understanding of salt tolerance of *A. ommaturus* at the molecular level and provide relevant transcriptome information for further study.

## Figures and Tables

**Table 1:** Summary of annotation of *A. ommaturus* unigenes.

Database	Number of Annotated Unigenes	Percentage of Annotated Unigenes
Nr	39,920	30.42%
KOG	21,947	16.72%
COG	11,807	9.00%
Pfam	27,697	21.11%
SwissProt	20,465	15.60%
KEGG	22,777	17.36%
GO	18,924	14.42%
At least one database	46,443	35.38%
Total unigenes	131,225	100.00%

**Table 2:** Representative osmotic pressure related genes involved in salinity stress.

Functional Group	Group	Gene Name	Gene Function	Gene ID	log_2_ (Fold Change)
Energy metabolism	0 vs. 15	glutathione S-transferase(rho)(*GST*)	transferase activity	96847_c4_g1	−1.117
		mevalonate kinase	isoprenoid biosynthetic process	94143_c2_g1	1.148
		insulin-like growth factor-binding protein 1(*IGFBP1*)	regulation of cell growth	78537_c0_g1	−1.489
		inositol-tetrakisphosphate 1-kinase-like	inositol trisphosphate metabolic process	94029_c1_g1	1.982
	15 vs. 30	Calreticulin (*CRT*)	protein folding	88942_c1_g2	−1.136
		transmembrane protease serine 9	proteolysis	95486_c1_g3	−1.234
		glutamine synthetase (*GS*)	glutamine biosynthetic process	85559_c6_g7	1.754
		phosphoenolpyruvate carboxykinase(*PEPCK*)	gluconeogenesis	89938_c3_g1	1.763
		glutathione S-transferase(rho)(*GST*)	transferase activity	96847_c4_g1	-1.907
		glutamate decarboxylase 1(*GAD1*)	carboxylic acid metabolic process	94253_c5_g1	-1.289
	15 vs. 45	glutamine synthetase (*GS*)	glutamine biosynthetic process	65875_c1_g1	1.141
		serine/threonine-protein kinase Sgk1	signal transduction mechanisms	87076_c4_g1	1.347
Ion transporters	0 vs. 15	sodium potassium ATPase beta (*NKA*)	potassium and sodium ion transport	92532_c3_g1	1.245
		transient receptor potential cation channel subfamily V member 4(*TRPV4*)	ion transmembrane transport	90898_c2_g1	1.341
		Aquaporin-3(*AQP3*)	water transport	94106_c4_g1	2.08
		FXYD domain-containing ion transport regulator 11(*FXYD11*)	ion transport	97290_c7_g1	−1.606
		sodium-coupled neutral amino acid transporter 2(*SNAT2*)	amino acid transport and metabolism	91967_c1_g2	−1.087
		cystic fibrosis transmembrane conductance regulator (*CFTR*)	chloride transmembrane transport	89496_c1_g1	−1.458
		Na^+^:K^+^:2Cl^−^ cotransporter 1a(*NKCC1a*)	Na^+^, K^+^, and Cl^-^ transport	92335_c1_g1	−1.004
	15 vs. 30	ferritin middle subunit	iron ion transport	90148_c4_g2	−1.265
		carbonic anhydrase 4(*CA4*)	metal ion binding	96156_c2_g1	1.480
		adenosylhomocysteinase 3 isoform X7	sodium ion transport	96455_c7_g1	1.007
		hemoglobin subunit α-1 (*HBA1*)	oxygen transport	84032_c0_g1	−1.764
		aquaporin-1 (*AQP1*)	water transport	82312_c0_g1	−2.372
	15 vs. 45	Na^+^/K^+^ ATPase α1b-ii (*NKA*)	potassium and sodium ion transport	93985_c4_g5	1.745
		sodium-coupled neutral amino acid transporter (*SNAT*)	amino acid transport and metabolism	91115_c3_g4	1.893
		aquaporin 3(*AQP3*)	water transport	94106_c4_g1	1.532
		FXYD domain-containing ion transport regulator 11(*FXYD11*)	ion transport	97290_c7_g1	1.791
		chloride channel protein 2(*CLCP2*)	chloride transmembrane transport	79986_c0_g1	−1.134
		Na^+^:K^+^:2Cl^−^ cotransporter 1(*NKCC1a*)	Na^+^, K^+^, and Cl^-^ transport	92335_c1_g1	1.495
		cystic fibrosis transmembrane conductance regulator (*CFTR*)	chloride transmembrane transport	89496_c0_g1	1.202
		FXYD domain-containing ion transport regulator 9(*FXYD9*)	ion transmembrane transport	95234_c8_g2	1.018
		sodium/potassium-transporting ATPase	potassium ion transport	85132_c6_g1	1.762
		ATP-sensitive inward rectifier potassium channel 15(*KCNJ15*)	potassium ion import	68888_c0_g1	1.829
Signal transduction	0 vs. 15	phosphatidylinositol 3-kinase regulatory	signal transduction	79932_c2_g2	1.092
		phospholipid phosphatase 3-like isoform X1	signal transduction	93118_c0_g1	1.062
		cAMP-responsive element modulator (*CREM*)	regulation of transcription	85301_c4_g2	−2.242
		cAMP-specific 3’,5’-cyclic phosphodiesterase 4B(*PDE4B*)	signal transduction	88103_c7_g1	−1.041
		prolactin receptor (*PRLR*)	prolactin signaling pathway	92757_c8_g1	2.672
	15 vs. 30	high affinity cAMP-specific and IBMX-insensitive 3’,5’-cyclic phosphodiesterase 8A	signal transduction	83321_c0_g1	−1.073
		putative transmembrane protein 116(*PTP*)	G-protein coupled receptor signaling pathway	85145_c0_g1	−2.122
	15 vs. 45	GTPase HRas	small GTPase mediated signal transduction	91669_c2_g4	1.139
		cAMP-responsive element modulator (*CREM*)	regulation of transcription	85301_c4_g2	1.089
		cyclooxygenase-2 (*COX2*)	inflammatory response	78208_c1_g1	1.132
		cAMP-specific 3’,5’-cyclic phosphodiesterase 4B(*PDE4B*)	signal transduction	90244_c2_g1	1.445
		Inositol monophosphatase(*IMP*)	signal transduction	94682_c12_g1	1.652
		MAP kinase-interacting serine/threonine-protein kinase 2-like	intracellular signal transduction	91691_c2_g1	1.333

## Supplementary Materials

The following are available online at https://www.mdpi.com/2073-4425/11/6/631/s1, Table S1: Primer sequences of the 8 target unigenes analyzed by qRT-PCR, Table S2: Summary of read statistics from RNA-sequencing of *A. ommaturus*, Figure S1: Top-hit species distribution, Figure S2: Proximal tubule bicarbonate reclamation, aldosterone-regulated sodium reabsorption, bile secretion, pancreatic secretion and mineral absorption.
