# Supplementary material for: Gill Transcriptome Sequencing and De Novo Annotation of Acanthogobius ommaturus in Response to Salinity Stress
Source: Genes (Basel). 2020 Jun 8;11(6):631. doi: 10.3390/genes11060631 (PMC7349121; doi:10.3390/genes11060631)
Supplement: Supplementary file 1 [file genes-11-00631-s001.pdf]

Table S1. Primer sequences of the 6 target unigenes analyzed by qRT-PCR.

Gene ID	Nr_Annotation	Primer (5′ to 3′)	PCR Product Length
\	β-actin	For_ TCGTGTGATTGAGCAACA<br>Rev_TAGACCACTACCAAGATTCG	100 bp
86284_c2_g1	Unknown	For_ GCTTCAAACAGTCCTTCA<br>Rev_ CCGTATCCACTACCACCA	148 bp
97290_c7_g1	FXYD domain-containing ion transport regulator 11 [*Kryptolebias marmoratus*]	For_ GGAGGCTTGGTTTTGTAA<br>Rev_ AAGGGAACTCTGTTGCAC	178 bp
90644_c1_g1	uncharacterized protein LOC109954007 [*Monopterus albus*]	For_ AAGGGAACAGGAGGGAAT<br>Rev_ ATAGGGTTGAGTGGGAGC	108 bp
88348_c0_g1	otopetrin-2-like [*Boleophthalmus pectinirostris*]	For_ GTTGTAGGTTGTGGCGTT<br>Rev_ ATGGAGTCTGTTTAGGCG	180 bp
85652_c0_g1	Uncharacterized protein LOC110162514 [*Boleophthalmus pectinirostris*]	For_ GCAGGTGAAAGTGGGTCT<br>Rev_ GTCATGGCAGGTGTAGTG	196 bp
85037_c0_g2	B-cell CLL/lymphoma 6 member B protein-like [*Boleophthalmus pectinirostris*]	For_ GGGAGACACAAGAGGAAA<br>Rev_ GGGAAAGACACTGAGGGA	174 bp
91346_c0_g1	Unknown	For_ CAGACACAGTTCAAAGCA<br>Rev_ ACCAAAGCCAGTCCACAT	134 bp
89909_c0_g1	complement C1q-like protein 2 [*Lates calcarifer*]	For_ ATTCAACTCTCACACCACTT<br>Rev_ GCTGATCGTTCTTCACCA	120 bp

Table S2. Summary of read statistics from RNA-sequencing of *A. ommaturus*.

Group	Raw Reads	Clean Reads	Error (%)	Q30 (%)	GC%
0-1	60,191,396	57,999,024	0.03	94.03	48.22
0-2	46,694,452	45,058,438	0.03	94.07	47.65
0-3	54,967,886	53,350,998	0.03	93.98	48.48
15-1	58,887,434	56,541,110	0.03	93.90	48.24

15-2	63,797,802	62,297,476	0.03	94.00	48.20
15-3	48,365,612	45,934,979	0.03	93.70	48.53
30-1	58,910,264	57,306,444	0.03	94.13	48.10
30-2	55,991,098	54,336,188	0.03	93.59	48.12
30-3	58,696,738	55,419,396	0.03	94.17	48.22
45-1	44,596,956	42,004,232	0.03	94.21	48.71
45-2	58,576,464	56,531,576	0.03	94.11	48.47
45-3	54,480,792	52,122,690	0.03	94.18	48.19

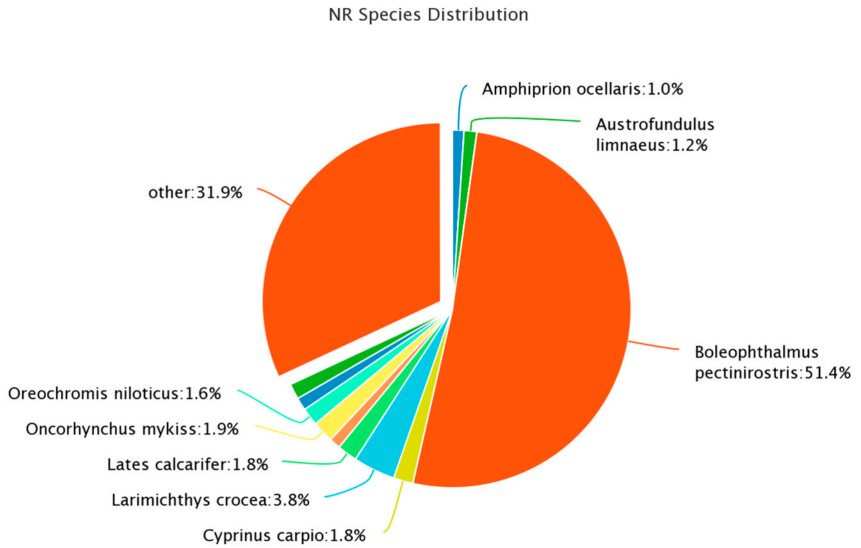

Figure S1. Top-hit species distribution.

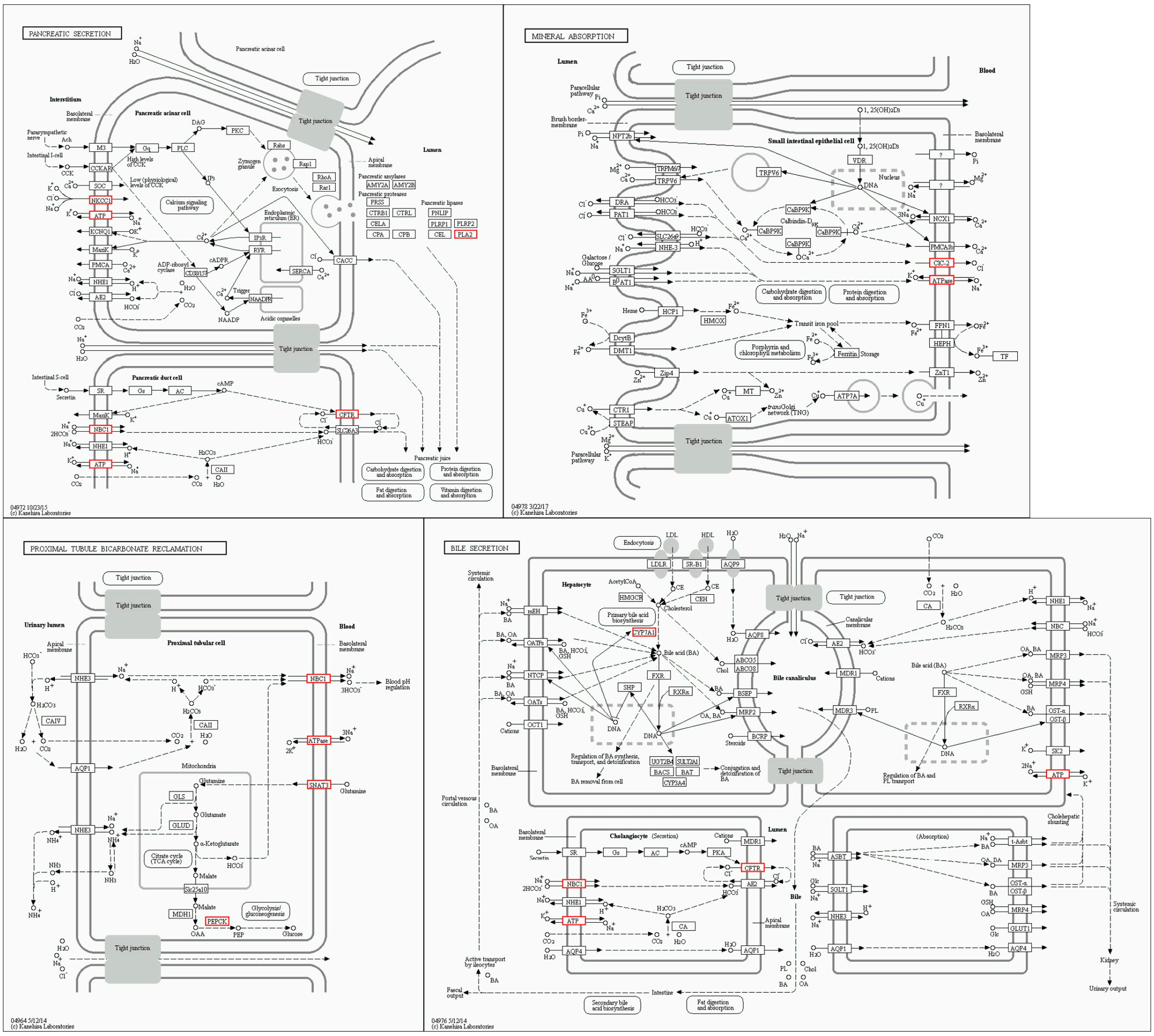

Figure S2. Pancreatic secretion, mineral absorption, proximal tubule bicarbonate reclamation and bile secretion.
